# An Ethical Framework for Evaluating Experimental Technology

**DOI:** 10.1007/s11948-015-9724-3

**Published:** 2015-11-14

**Authors:** Ibo van de Poel

**Affiliations:** Section Ethics and Philosophy of Technology, Department of Values, Technology and Innovation, Delft University of Technology, Jaffalaan 5, 2628 BX Delft, The Netherlands

**Keywords:** Technology, Experiment, Ethics, Bioethical principles, Human subjects, Informed consent

## Abstract

How are we to appraise new technological developments that may bring revolutionary social changes? Currently this is often done by trying to predict or anticipate social consequences and to use these as a basis for moral and regulatory appraisal. Such an approach can, however, not deal with the uncertainties and unknowns that are inherent in social changes induced by technological development. An alternative approach is proposed that conceives of the introduction of new technologies into society as a social experiment. An ethical framework for the acceptability of such experiments is developed based on the bioethical principles for experiments with human subjects: non-maleficence, beneficence, respect for autonomy, and justice. This provides a handle for the moral and regulatory assessment of new technologies and their impact on society.

## Introduction

Swarm robots. Human enhancement. Algae based on synthetic biology. Automated driving vehicles. What these new technological possibilities have in common is that they may seriously impact society, for the good as well as for the bad. What they also have in common is that the exact impacts on society are currently largely unknown and are very hard to predict beforehand. As a consequence, the current moral and regulatory appraisal of such technologies is often either based on what we know and can scientifically prove (in so-called science-based or evidence-based approaches) or on scenarios that might occur but of which the probability is unknown (in so-called precautionary approaches). Both types of approaches, however, run a risk of missing out on important actual social consequences of new technologies and of making us blind to surprises. Therefore, both approaches do not really address the uncertainty that is inherent in the introduction of new technology into society.

In this respect, Jasanoff speaks of ‘technologies of hubris’, i.e. those “predictive methods (e.g., risk assessment, cost-benefit analysis, climate modelling) that are designed, on the whole, to facilitate management and control, even in areas of high uncertainty” (Jasanoff 2003: 238). Such predictive methods, however, have three shortcomings according to her. First, they deny uncertainty and ignorance; second, they short-circuit the moral dimension of new technological developments; third, they do not address the need for profound (social) learning from, for example, errors and catastrophes. As an alternative, she proposes the development of what she calls ‘technologies of humility,’ which address issues of framing, vulnerability, distribution, and learning.

This article aims to contribute to what Jasanoff calls technologies of humility. It does so in two ways. First, I will propose a conceptualization of new technology as a kind of social experiment that stresses the experimental character of new technology and the role of uncertainty and ignorance, and the need for learning. Second, on basis of that conceptualization and (bio-ethical) literature on the acceptability of experiments with human subjects, I will develop an ethical framework for evaluating the introduction of such experimental technologies into society. This framework among others addresses issues of what Jasanoff calls vulnerability and distribution.

It must be admitted that both my contributions are not entirely new, as they build on the work of many others as well as some of my own previous work. In particular, the ethical framework, I will present is very similar to that presented in van de Poel (2011). As suggested there and by Robaey and Simons (2015), this ethical framework can be derived from the bioethical principles of non-maleficence, beneficence, respect for persons, and justice. In these earlier publications, however, the relation between the conditions in the framework and the bioethical principles is not worked out in as much detail, as it is done here.

This article starts with a brief discussion of the control dilemma in technological development as a background for the difficulties of dealing with experimental technologies; it then sketches the experimental introduction of new technology into society as an alternative to deal with the control dilemma. After, this conceptualization I focus on the question under what conditions such experiments with new technology in society are morally acceptable. I first discuss the principle of informed consent that has been proposed by Martin and Schinzinger (1983, 1996) to deal with the moral acceptability of such experiments but I find it lacking, and argue that we should approach the issue from a broader set of moral principles. I then take on the task to specify the bioethical principles of non-maleficence, beneficence, respect for autonomy, and justice for experimental technologies. To do so, I first sketch how these principles underlie more concrete moral requirements that have been formulated for clinical experiments and then I use this as a starting point to formulate more specific moral conditions for the introduction of experimental technology into society.

## The Control Dilemma: Anticipation Versus Incrementalism

I will call technologies experimental if there is only limited operational experience with them, so that social benefits and risks cannot, or at least not straightforwardly, be assessed on basis of experience. Of course, there are other ways of assessing risks and benefits of such technologies including simulation and lab experiments and, more recently, so-called living labs. Still, the introduction of such technologies into society comes with large uncertainties, unknowns and indeterminacies that are often only reduced once such technologies are actually introduced into society. Given these uncertainties and unknowns, the introduction of such technologies into society can be conceived as a social experiment.

According to this line of thinking, building a new bridge of a known design would not be experimental while the introduction of Google Glass into society would surely be experimental. Of course, the question where to draw the line between experimental and non-experimental technology is not easy to answer. Above I have suggested that operational experience is an important factor but how much and for how long a period, operational experience is required may well depend on the technology and the kind of (social) impacts one is interested in or worried about. There is now more than fifty years of operational experience with nuclear energy which makes this technology no longer experimental in some respects, but this experience is arguably still very minor when it comes to the issue of nuclear waste disposal, which is to be stored safely for periods up to 10,000 years.

The difficulties in dealing with experimental technologies go back to the control dilemma that was formulated by David Collingridge (1980). This dilemma says that in the early phases of new technology, when a technology and its social embedding are still malleable, there is uncertainty about the social effects of that technology. In later phases, social effects may be clear but then often the technology has become so well entrenched in society that it is hard to overcome negative social effects.

In the past decades, most approaches have tried to overcome the first horn of the control dilemma by improving the anticipation of the consequences of new technology. The aim was to reduce the uncertainty in the early phases of technological development so that technologies can be pro-actively designed to avoid possible negative consequences and risks and to attain positive effects and values. This is the approach in, for example, Constructive Technology Assessment (Rip et al. 1995), Value Sensitive Design (Friedman et al. 2006) and in Responsible Innovation (Owen et al. 2013). A similar emphasis on anticipation can be found in the ELSI (Ethical, Legal and Social Implications) programs that accompany the Human Genome Project and the National Nanotechnology Initiative in the USA, and in recent RRI (Responsible Research and Innovation) initiatives as part of Horizon 2020 in Europe.

While the goal of early anticipation of the social consequences of new technology is laudable, anticipation has its limits. Anticipation will usually not reduce all unknowns and surprises are still likely to occur (cf. also Gross 2010). Moreover, anticipation may well lead to a focus on scenarios that are morally thrilling but very unlikely, like the famous grey goo scenario in nanotechnology that was developed by Eric Drexler (1986). In the grey goo scenario, small nanorobots get out of control and become self-replicating and they then consume all the matter on the planet. While the grey goo scenario is not entirely impossible, even Drexler has admitted that it is not very likely and that other social and ethical concerns with respect to nanotechnology deserve more attention (Phoenix and Drexler 2004). The problem of these kinds of scenarios is not just that they are unlikely but also that they draw moral and regulatory attention away from the more important ethical issues in fields like nanotechnology (Nordmann 2007).

There is, however, an alternative to anticipation as a way to address the control dilemma. This alternative is the gradual and experimental introduction of a technology into society, in such a way that emerging social effects are monitored and are used to improve the technology and its introduction into society. This alternative approach goes back to the work of such thinkers as the political scientist and economist Charles Lindblom, and philosophers like Karl Popper and John Dewey.

Popper (1945) has argued for what he called piecemeal social engineering, rather than revolutionary social change. Such an approach to social issues, which can also be applied to the introduction of technology into society, is based on social experiments in a limited part of society and on learning from experience and error.

Lindblom is known as an opponent of comprehensive rational (large-scale) planning and he placed much emphasis on incrementalism and the importance of trial-and-error learning. He emphasized that due to our limited information-processing capacities and due to uncertainties and unknowns, we can usually not plan rationally but have ‘to muddle through’ (Lindblom 1959). The best we can do often is to proceed in small or limited steps and to learn from trial and error. These ideas have been further developed and applied to technology by authors like Collingridge and Woodhouse (Morone and Woodhouse 1986; Collingridge 1992; Woodhouse and Collingridge 1993). Collingridge (1992), for example, stresses the importance of trial-and-error learning, incremental decision-making, and flexibility and adaptability, and shows how a number of costly technical failures are due to a lack of such an approach. Also the work of Wildavsky on dealing with technical risks is relevant. Wildavsky (1988) argues that attempts to anticipate and prevent risks often come at the costs of the ability to deal with unexpected risks and surprises, which he understands in terms of resilience.

## New Technologies as Social Experiments

One way to further conceptualize the experimental introduction of a technology into society is to conceive of the introduction of new technology into society as a kind of social experiment. One of the first publications that proposed the idea of new technologies as social experiments was an article by Krohn and Weyer (1994) published in *Science and Public Policy*. They do not speak about social experiments but about real-world experiments, and mainly focus on the unpredictability of risks of new technologies.

A somewhat similar idea was already proposed in engineering ethics in the textbook *Ethics in Engineering* by Martin and Schinzinger, of which the first edition appeared in 1983 (Martin and Schinzinger 1983). They speak of engineering as a form of social experimentation for quite similar reasons as Krohn and Weyer talk about real-world experiments, and they propose the principle of informed consent to deal with the acceptability of such experiments.

A 2007 report by the *European Expert Group on Science and Governance* again stressed the importance of the notion. As they noted, “we are in an unavoidably experimental state. Yet this is usually deleted from public view and public negotiation” (Felt et al. 2007: 68) And they continue: “If citizens are routinely being enrolled without negotiation as experimental subjects, in experiments which are not called by name, then some serious ethical and social issues would have to be addressed” (Felt et al. 2007: 68).

The idea of the introduction of a new technology as a form of social or real-world experimentation has been applied to several domains and cases including waste facilities (Herbold 1995), urban studies (Gieryn 2006), regulation (Millo and Lezaun 2006), genetically modified crops (Levidow and Carr 2007), engineering research laboratories (Fisher and Lightner 2009), ecological restoration (Gross and Hoffmann-Riem 2005; Gross 2010; Schwartz 2014), sunscreens with nanoparticles (Jacobs et al. 2010), nuclear power (Krohn and Weingart 1987; Van de Poel 2015), sustainable development (Böschen 2013), nature conversation (Lorimer and Driessen 2014), and even to the creation of a European identity through the development of new technologies and a European science and technology policy (Nordmann 2009).

The general idea of experimentation in the real-world is already older than its use in the domain of technology. Main forerunners were pragmatist philosophy, in particular the work of John Dewey, and the *Chicago School of Sociology* in the late 19th and early 20th century, in particular the work on urban studies (see Gross and Krohn 2005; Hutchison 2010). Dewey argued for applying the experimental method not only to social science but also to politics and ethics. He speaks of the formation of states as an experimental process (Dewey 1927: 32), and calls the introduction of policy measures a kind of experiment (Dewey 1938: 508–509). He also believes that ethics is, or at least should be, experimental. Moral principles are not unchangeable prescripts but rather hypotheses to be tested out in new situations (Dewey 1922: 239).

Around the same time as Dewey, sociologists from the Chicago School began to speak of social experiments as experiments that do not take place in the laboratory but in the real-world. In fact, such social experiments could be found everywhere in society. As Albion Small expressed it: “All the laboratories in the world could not carry on enough experiments to measure a thimbleful compared with the world of experimentation open to the observation of social science. The radical difference is that the laboratory scientists can arrange their own experiments while we social scientists for the most part have our experiments arranged for us.” (Small 1921: 188) Robert Park spoke of the city as a social laboratory, an idea that become quite influential in urban studies (Park 1929).

Whereas in the Chicago tradition of sociology, social experiments in the real-world were not seen as a derivate version of traditional laboratory experiments (see Gross and Krohn 2005; Gross 2009), this was somewhat different in a later tradition that emerged in the United States, especially in relation to the “Income Maintenance Experiments” that were carried out between 1968 and 1982. Here social experiments were as much as possible set up as randomized trials or as quasi-experiments (Campbell and Stanley 1966), so that there was a control group to establish the effects of certain policies in a systematic and comparative way. Campbell was one of the main proponents of this movement (see e.g. Campbell and Russo 1999).

More recently, the idea of experimentation has also been taken up in public administration and in law under the name of ‘democratic experimentalism’ (see e.g. Butler 2012). This development was fueled by an article by Dorf and Sabel in 1998 in which they proposed a “Constitution of Democratic Experimentalism” for the US, inspired by the work of John Dewey (Dorf and Sabel 1998). It has been argued that also in Europe a trend towards more experimental governance is visible (Sabel and Zeitlin 2010). Notions like adaptive management and adaptive governance also seem to fit in this development (Ansell 2012).

## Towards an Ethical Framework for Experimental Technology: Informed consent

When a new technology is introduced into society it amounts to a de facto social experiment because even if all reasonable efforts to anticipate social consequences haven been undertaken, it is possible, and even likely that there will be unanticipated social consequences. This de facto experimentation can be turned into a mode of more deliberate and responsible experimentation, for example, by following Popper’s idea of piecemeal social experiments. Such responsible experimentation needs to meet both epistemological and ethical constraints. Epistemological constraints are important to ensure learning from social experiments. Ethical constraints are important because these experiments take place in society and may seriously harm individuals as well as society as a whole.

Martin and Schinzinger (1983, 1996) have proposed *informed consent* as a main ethical principle to judge the moral acceptability of social experiments with new technology. The application of this principle to such experiments is, however, problematic. First, it may be very hard to identify all individuals that are potentially affected by the introduction of a new technology into society (even if it happens only in a part of society) and to ask them for their informed consent. And even if this would be possible, it is sometimes questionable whether it is ethically desirable because it would give each individual that is affected a veto power however large the benefits to society (Hansson 2004). This problem is due to the fact that whereas in medicine, and in clinical experiments, risks are usually borne individually, in technology risks may be individual as well as collective. Whereas risks from for example nanoparticles in sunscreens or electromagnetic emissions from mobile phones are largely individual, the risks of nuclear melt-down or of an explosion in a chemical plant are collective.

To deal with this problem, Martin and Schinzinger propose the following specification of informed consent for situations in which individuals cannot be readily identified:

“Information that a rational person would need, stated in understandable form, has been widely disseminated.

The subject’s consent was offered in a proxy by a group that collectively represents many subjects of like interests, concerns, and exposure to risk” (Martin and Schinzinger 1996: 87).

It remains unclear, however, whether they understand the second condition to require unanimous consent by the representative group or only a majority decision. In the first case, the requirement of informed consent might be too strict as I argued above. In the second case, it may be doubted whether what they propose is still a form of informed consent or rather another specification of the broader principle respect for autonomy on which informed consent is based (and which may be ethically justifiable in its own right as I will argue below).

Also the first condition proposed by Martin and Schinzinger is problematic in a technological context, especially for experimental technology on which I focus here. Risks and benefits of experimental technologies may not only be hard to estimate and quantify, sometimes they are unknown. It seems that the “information that a rational person would need” to give informed consent is sometimes simply not available in the case of experimental technology. Again, this is a difference with medicine and clinical experiments, where usually risks are better known, or at least the possible effects are known even if probabilities may not always be reliably known (cf. Asveld 2006).

The above argument points at differences between technology and medicine that make it harder, if not impossible to apply the principle of informed consent to experimental technology. But even for clinical research, it has been argued that informed consent is neither a sufficient nor a necessary condition for the acceptability of experiments involving human subjects (Emanuel et al. 2000). So rather than focusing on informed consent, it would be advisable to focus on the broader and more encompassing set of moral principles that have been articulated in the literature on ethics of experiments with human subjects (including clinical experiments) and to see how these would apply to social experiments with technology. This provides for a broader approach than just a focus on informed consent. Rather than trying to apply the informed consent principle to experimental technologies, we look for the underlying moral principle (respect for autonomy in this case^1^) and we see how this can best be specified in the context of experimental technology.

## Developing an Ethical Framework

The argument above was that the principle of informed consent is both too specific and too narrow to be a good basis for an ethical framework for evaluating the introduction of experimental technology into society. Therefore, I propose to start from the broader and more general set of ethical principles that have been articulated in bioethics: non-maleficence, beneficence, respect for autonomy, and justice (Beauchamp and Childress 2013). These principles have been specified in terms of more specific moral principles and rules in the context of medical experiments and other experiments with human subjects. However, these more specific interpretations suppose a context that is different from that in which new technologies are introduced into society. For example, in the technological context, it is often harder to identify individual human subjects and risks may be more uncertain or even unknown. Therefore, in the context of technological experiments in society, we need to develop a new specification of these general moral principles.

The four principles of non-maleficence, beneficence, respect for autonomy, and justice have been particularly articulated by Beauchamp and Childress (2013).^2^ Other authors have proposed other ethical principles for clinical experiments. For example, Emanuel et al. (2008) mention the following eight principles for judging the acceptability of clinical experiments: collaborative partnership, social value, scientific validity, fair participant selection, favorable risk–benefit ratio, independent review, informed consent, and respect for participants. They claim that these principles are individually necessary and jointly sufficiently to establish the acceptability of a clinical experiment (Emanuel et al. 2008: 132).^3^

The reason I do not focus on these eight principles is that I believe they are too context-specific to be a good basis for making the translation to the context of experimental technology. As I have pointed out this context is different in terms of the nature of the risks (not just individual but also collective risks), and the degree of knowledge of the risks (not just uncertain but also unknown risks).

I also believe that the eight principles of Emanuel et al. (2008) can be understood and justified in terms of the four principles of non-maleficence, beneficence, respect for autonomy, and justice. For example, scientific validity can be understood in terms of beneficence. As Emanuel et al. (2008: 127) point out doing scientifically adequate experiments is not just important for scientific reasons but for ethical reasons as well: “Valid science is a fundamental ethical requirement”. The requirement guarantees that the experiment produces knowledge and so has an added value for society, which is clearly related to, and can be justified in terms of beneficence.

Table 1 indicates how in my view the eight principles of Emanuel et al. (2008) are related to the four principles of Beauchamp and Childress (2013). If we compare my interpretation with a similar exercise in Emanuel et al. (2000), which discusses 7 of the 8 principles in Emanuel et al. (2008), two differences come to the fore. First, I have not added nonexploitation as an additional ethical value or principle. The reason is that I believe that nonexploitation is more an ethical value underlying the four principles of non-maleficence, beneficence, respect for autonomy, and justice than an additional principle at the same level (see also Emanuel et al. 2008: 125). Nonexploitation indeed seems related to the fulfillment of all four moral principles. Informed consent, or respect for autonomy, may be required to avoid exploitation, but informed consent will usually not be enough to avoid exploitation (Wertheimer 2008); it will also require some conditions of non-maleficence and justice, and possibly also of beneficence to be fulfilled. Nonexploitation then remains important as an underlying value but I don’t think it needs to be added to the four moral principles of Beauchamp and Childress. A second difference is that Emanuel et al. (2000) list accountability and minimizing the influence of potential conflicts of interest as an ethical value behind independent review while I have interpreted that in terms of ‘procedural justice’, which I take to be a part of the moral principle of justice.

**Table 1:** Relation between ethical principles for clinical experiments (Emanuel et al. 2008) and bioethical principles (Beauchamp and Childress 2013)

Principle (Emanuel et al. 2008)	Bioethical principle (Beauchamp and Childress 2013)
Collaborative partnership	Justice, respect for autonomy, non-maleficence
Social value	Beneficence
Scientific validity	Beneficence
Fair participant selection	Justice
Favorable risk–benefit ratio	Beneficence, non-maleficence
Independent review	(Procedural) justice
Informed consent	Respect for autonomy
Respect for participants	Respect for autonomy, justice, non-maleficence

## The Ethics of Experimentation

Before I specify the four general moral principles for experimental technology, I first will elaborate a bit more how these principles have been specified for clinical experiments. To do so, I looked at three main codes in the domain of clinical experimentation and experiments with human subjects: the Nuremberg code, the Helsinki Declaration and the so-called Common Rule in the US (in particular its codification in the US Code of Federal Regulations, Title 45 (Public Welfare), Part 46 (Protection of Human Subjects)). For each code, I related the articles in the code to one (or more) of the four bioethical principles.

The goal of this exercise was twofold. Firstly, it was meant to check whether it is indeed the case, as I claimed above, that the four bioethical principles cover all, or at least most, of the moral concerns and conditions that have been worded in these codes. Second, this exercise was meant to come to a number of more or less commonly accepted specifications of the principles for clinical experiments. Of course, for reasons that I have explained above these specifications cannot be directly applied to the context of experimental technology. Still, they provide a good starting point for the specification of the four principles also in the domain of experimental technology. As we will see below, sometimes the specifications could more or less directly be translated from the medical to the technological domain. In other cases, the specific conditions did no longer meaningfully apply to the context of experimental technology. But in such cases, we should be able to give reasons why it no longer applies and on basis of these reasons we can decide whether the condition can maybe be left out in the context of experimental technology because the underlying moral concerns do not longer apply in that context or require another specification because the moral concern is still relevant but needs another specification due to the new context.

The exercise was done on the latest version of the three codes. The Nuremberg Code has not been reformulated since its formulation in 1949, but the other two have regularly been revised. I looked at the 2009 version of the Common Rule and the 2013 version of the Helsinki Declaration. For the Common Rule, I only included article §46.111 “Criteria for IRB [Institutional Review Board] approval of research” in the analysis because the other articles are more explanatory or procedural in nature or they are a further specification of the articles in §46.111, and these further specifications seemed to be too specific for the current purpose.^4^

The coding was done by reading through the codes and by coding each article with one or more of the following terms:

Non-maleficence: Obligations relating to doing no harm, including obligations to minimize risks, or to take precautions against possible risks or harms from the experiment
Beneficence: Obligations to do good, including obligations to take away existing harm, or to prevent harm or risks that do not originate in the experiment,^5^ to produce more good than harm, to create or increase benefits
Respect for autonomy: Obligations relating to protecting and guaranteeing the autonomy, including the autonomous choice, of individuals and groups
Justice: Obligations relating to issues of distributive justice, to special protection of vulnerable groups, to avoiding exploitation, but also to procedural justice (just procedures)^6^

An article was coded with one of the above terms if the article either exemplified this term, or as the term could be seen as the motivation or justification for the obligations worded in the article.

It turned out that almost all articles in the codes could be coded with at least one of the moral principles and that these moral principles in these cases more or less covered all the obligations stated in the article. In cases, in which one moral principle did not cover the specific obligations stated in the article, another moral principle was added until all obligations were covered. Only for two types of cases, the obligations worded in the article were not, or not completely, covered by one or a combination of the four bioethical principles. First, there were some articles that did not really contain normative obligations but which rather contained background information or an explanation of the code; these were coded as “explanation”. Second, in two instances it turned out that that not all moral obligations were covered. In both cases, reference was made to duties and responsibilities of a specific group. These were coded as “responsibility”:

Responsibility: Indicates that a specific group or person has a duty or responsibility with respect to a certain moral obligation

It should be noted that this principle of responsibility does not add substantial moral obligations to the ones covered by non-maleficence, beneficence, respect for autonomy and justice. Rather it specifies *who* has a duty or is responsible for living by or upholding these moral obligations. So while responsibility adds a moral dimension that is not covered by the four bioethical moral principles, it does not add substantive moral obligations not covered by the four principles. By and large, then, the coding exercise corroborated the hypothesis worded above that the four bioethical principles cover the moral obligations relating to experiments with human subjects (at least if the three discussed codes are taken as covering the relevant moral obligations).

When we look in more detail at which principles are specified in each of the three codes, it strikes one in the eye that the Nuremburg Code does not contain specific moral obligations relating to the principle of justice. There seem to be two, connected, historical explanations for this. First, the Nuremberg Code was formulated in response to the atrocities of World War Two and Nazi experiments on human beings. This probably explains why the code places most emphasis on respect for autonomy (informed consent in particular) and non-maleficence, and to a lesser degree beneficence. The other explanation is that the code was never revised like the other two codes. It seems that the principle of ‘justice’ has received more attention in the course of time in the ethics of human experimentation. This suggests, interestingly, that not only the specification of the four principles is a dynamic process, as testified by the regular revisions of the Helsinki Declaration and the Common Rule, but that even what are seen as the underlying principles might develop over time.^7^

After coding the articles in the three codes, I grouped together articles that contained more or less similar specific moral obligations. Table 2 is the result of this exercise. Under each of the four bioethical principles, and the additional one of responsibility, it lists a number of more specific obligations that can be found in the three analyzed codes, and it indicates the articles from the codes that contain this specific obligation. Sometimes, an article contained more than one more specific obligation so that it appears more often than once in the list. The resulting more specific obligations, as listed in Table 3, were the starting point for specifying the bioethical principles, and the principle of responsibility, in the context of experimental technology.

**Table 2:** Specification of the moral principles for clinical experiments that can be found in the Nuremberg Code (NC), Helsinki Declaration (HD) and Common Rule (CR)

Moral principle	Specific rules and considerations	Codes	Conditions in Table 3
Non-maleficence	1a. No other means for acquiring knowledge (first other modes of acquiring knowledge)	NC2	1
	1b. Monitoring of data and of risks	CR6, HD17	2
	1c. Possibility and willingness to adapt or terminate the experiment	NC10, HD18	3
	1d. Avoid unnecessary harm, minimize risks	NC4, CR1, HD14, HD17, HD28	4, 5, 6, 7
	1e. Avoid death or disabling injury	NC5	4, 5, 6, 7
	1f. Measures to protect against possible risk	NC7, HD17	4, 5, 6, 7
	1g. Minimize harm to the environment	HD11	4, 5, 6, 7
	1h. Protect privacy of experimental subjects	CR7, HD24, HD32	2
Beneficence	2a. Benefits to society	NC2	8
	2b. Anticipated results justify performance of experiment	NC3	8
	2c. Positive benefit/risk ratio	NC6, CR2, HD16	n.a.
	2d. Qualified experimenters	NC8, HD12	9
	2e. Scientifically and methodologically sound	NC3, HD21	2, 4
	2f. Publication and dissemination of outcomes of study	HD36	n.a.
	2g. Access to unproven but hopeful intervention if no effective intervention available	HD37	n.a.
	2h. Physician should promote and safeguard the health, well-being and rights of patients	HD3, HD4, HD7	n.a.
Respect for autonomy	3a. Informed consent	NC1, CR4, HD 25–32	10, 11, 12
	3b. Possibility to withdraw from the experiment	NC9, HD29, HD31	13
Distributive justice	4a. Equitable selection of subjects	CR3	14
	4b. Protect vulnerable subjects	CR8, HD19	14
	4c. Only vulnerable groups if they also profit from the research	HD20	15
	4d. Appropriate access to experiment and results for underrepresented or vulnerable groups	HD13, HD20, HD28	15
	4e. Test against best proven intervention	HD33	15
	4f. Post-trial access to intervention for all experimental subjects	HD34	15
	4g. Rights and interests of research subjects are more important than knowledge acquisition	HD8	15
	4h. Compensation and treatment for harm	HD15	16
Procedural justice	5a. Documentation of informed consent	CR5	n.a.
	5b. Presence of adequate research protocol	HD22	9
	5c. Ethics committee/IRB	HD23	11
	5d. Registration in publicly available database	HD35	n.a.
Responsibility	6a. The duty and responsibility for ascertaining the quality of the consent rests upon each individual who initiates, directs or engages in the experiment	NC1	n.a.
	6b. Physician remains responsible for health of patients even if informed consent has been given	HD9	n.a.

**Table 3:** An ethical framework for experimental technology

1	Absence of other reasonable means for gaining knowledge about risks and benefits
2	Monitoring of data and risks while addressing privacy concerns
3	Possibility and willingness to adapt or stop the experiment
4	Containment of risks as far as reasonably possible
5	Consciously scaling up to avoid large-scale harm and to improve learning
6	Flexible set-up of the experiment and avoidance of lock-in of the technology
7	Avoid experiments that undermine resilience
8	Reasonable to expect social benefits from the experiment
9	Clear distribution of responsibilities for setting up, carrying out, monitoring, evaluating, adapting, and stopping of the experiment
10	Experimental subjects are informed
11	The experiment is approved by democratically legitimized bodies
12	Experimental subjects can influence the setting up, carrying out, monitoring, evaluating, adapting, and stopping of the experiment
13	Experimental subjects can withdraw from the experiment
14	Vulnerable experimental subjects are either not subject to the experiment or are additionally protected or particularly profit from the experimental technology (or a combination)
15	A fair distribution of potential hazards and benefits
16	Reversibility of harm or, if impossible, compensation of harm

## Conditions for Responsible Experimentation in the Context of Experimental Technology

### Non-maleficence

A first moral principle is non-maleficence, which means that one ought not to (intentionally) inflict evil or harm (Beauchamp and Childress 2013). Harm is here understood as an adverse effect on somebody’s interest. The problem of applying this moral principle is that social experiments with new technology by their very nature involve the possibility of unknown harm. Therefore we cannot simply require that no harm will ensue. Still, we can require that harm is prevented as far as reasonably possible and that, if harm occurs, either the experiment is stopped or that measures are taken to avoid or at least reduce harm.

The conditions 1 through 7 in Table 3 can be seen as a specification of the principle of non-maleficence for social experiments with technology. Condition 1 requires that before a technology is introduced into society (as a form of social experimentation), first all other reasonable means to gain knowledge about possible risks of the technology, like lab tests or field tests, have been exhausted. This is similar to condition 1a in Table 2. Conditions 2 and 3 require that the experiment is monitored (condition 2), and that if harm occurs the experiment can be stopped or can be adapted to avoid or minimize harm (conditions 3). These conditions are similar to the conditions 1b and 1c for clinical experiments. Condition 1 h from Table 2 about privacy protection has been included in condition 2 because it seems more a subcondition to condition 2 than a requirement itself for responsible experimentation with new technologies.

Condition 4 states that harm should be contained as far as is reasonably possible. This obligation is similar to the obligations worded in the conditions 1d through 1 g in Table 2. For reasons explained above, a complete avoidance of harm—or of certain specific harms as suggested in condition 1e for clinical experiments—is usually not possible for experimental technologies.

The conditions 5 through 7 all aim at achieving non-maleficence through the strategy of incrementalism (rather than anticipation) that was explained above. Condition 5 follows from Popper’s (1945) idea of piecemeal social experiments and is intended to avoid large-scale harm and to increase what is learned from the experiment. Condition 6 is based on the idea of Collingridge (1992) that incrementalism requires flexibility, in order to be able to deal with the control dilemma. This can be further extended to include the avoidance of what has been called the lock-in into a technological option, so that other technological options are no longer considered or it has become much harder to switch to other technologies (Arthur 1989; see also Bergen’s contribution in this special issue). Condition 7 follows from Wildavsky’s (1988) idea that in order to deal with the risks of new technology we should not solely depend on containment of expected risks, but also on resilience in order to be able to deal with unknown or unexpected risks.

### Beneficence

The moral principle of beneficence says that we should not only avoid harm but also (seek to) do good. The importance of this principle in the medical context is quite obvious as medicine is expected to contribute to human health and, ultimately, to human well-being. Some might want to argue that it is not obvious that beneficence is also relevant in a technological context. One could argue that companies and other actors should be free to develop and introduce new technologies into society as long as they do not harm others (non-maleficence).

The point of experimental technologies is, however, that there is always the possibility of unknown harm. Introducing such possible but unknown harm would seem only be permissible if it is reasonable to expect at least some benefits from the experiment. This is what is expressed in condition 8.

For experimental technologies, we often do not know the potential benefits and drawbacks well enough to list all possible effects and to assign probabilities. Therefore condition 8 is formulated in terms of whether it is reasonable to expect social benefits from the experiment, which is similar to condition 2a in Table 2, rather than in terms of the (overall) balance or ratio of benefits and risks. The reason is that balancing risks and benefits requires rather accurate knowledge of risks and benefits (including their magnitude) and the point of experimental technology is that such knowledge is usually lacking. It therefore seems better to use a criterion that requires less anticipatory knowledge of social impacts. For similar reasons, no equivalent to condition 2b from Table 2 was specified. Moreover, in as far as consequences can be anticipated this condition seems to be largely covered by condition 8.

Condition 9 was developed as an alternative specification of the conditions 2d and 2e and the principle of responsibility in Table 2. A first thing to be noted here is that conditions 2d and 2e for clinical experiments suppose scientific experiments with experimenters that can be clearly delineated. This assumption no longer holds for the case of experimental technology. Here we are dealing with social experiments that do not have a clearly distinguishable, scientifically trained experimenter. Rather, these experiments are done by practitioners in society, or by a range of actors like engineers, companies, governments, or maybe even by society.

Still, we should address the underlying moral concern that is specified in conditions 2d and 2e in Table 2. This concern is that something is learned from the experiment that benefits society. In this case, the learning, however, is not scientific learning through hypothesis testing but rather a kind of trial-and-error learning about an on-going intervention through the experimental introduction of a technology into society. This learning is enabled by some of the already mentioned conditions like condition 2 (monitoring) and condition 5 (gradually scaling up to enable learning). But given the fact that there is not one experimenter, learning also requires a clear distribution of responsibilities among the various actors, as worded in condition 9.

Condition 9 can also be seen as a specification of the moral principle of responsibility. Conditions 6a and 6b for clinical experiments do not directly apply to the context of experimental technology as they relate to informed consent and single out persons (doctors, experimenters) that are not directly relevant in the new context. Still, the principle of responsibility is relevant for experimental technology, and maybe even more relevant than in the clinical context. Although technology will often be introduced in institutional settings with some predefined responsibilities, these do not necessarily reflect the idea that introducing new technology amounts to a social experiment; moreover responsibilities are often shared by multiple actors and may not, or unclearly, be distributed over these actors.

### Respect for Autonomy and Justice

Conditions 10 through 13 are intended to safeguard the moral principle respect for autonomy, and can be seen as an alternative to the principle of informed consent that is often not directly applicable to the context of experimental technology as I have argued above. Condition 10 covers the ‘informed’ part of informed consent. But rather than requiring individual consent, condition 11 requires a form of collective consent by approval by a democratically legitimized body. A potential problem of such collective consent is that it may lead to a tyranny of the majority, requiring unacceptable sacrifices from individuals for the collective good. Conditions 12 and 13 and the conditions 14 through 16, which address the moral principle of justice, can be seen as a way to avoid such exploitation. They guarantee that experimental subjects have a say in the set-up of the experiment (condition 12), and are able to withdraw from the experiment (condition 13); the latter condition is similar to condition 3b in Table 2.

They also guarantee that vulnerable people are either additionally protected or are not subjected to the experiment (condition 14) and that risks (and/or other costs) and benefits are fairly distributed (condition 15), so that certain groups do not bear all the burdens without having any benefits. The last two conditions are especially important in the light of the moral principle of justice. Condition 14 is indeed similar to conditions 4a and 4b in Table 2. Conditions 4c through 4 g in Table 2 can all be understood as setting some minimal conditions for the just distribution of benefits and risks among the involved groups. In the case of clinical experiments usually three main groups can be distinguished: the experimental group undergoing the intervention, the control group (undergoing another intervention or no intervention), and the larger population that might profit from the results (including vulnerable groups within this larger population). In the case of technologies, risks and benefits may be distribution over a larger number of groups and distribution effects may be more complicated. While in medicine the main effects are health effects for individuals, some technologies may also shift the power relations between groups and so have complicated distribution effects. Rather than the quite specific principles 4c through 4 g, for experimental technology a much more general condition has been formulated as condition 15.

The final condition for justice worded in condition 4 h in Table 2 has been translated into condition 16 that states that, if possible, irreversible harm should be avoided, which can be seen as a specification of non-maleficence and when irreversible harm nevertheless occurs, compensation should be offered

## Status of the Conditions

The conditions listed in Table 3 are a first attempt to specify conditions for experimental technology on the basis of four bioethical moral principles taking their current specification for clinical experiments as an inspiration. The new specification was done by looking at experimental technology in general rather than by focusing on specific experimental technologies. Arguably, the specification may need to be somewhat revised or further specified for specific technologies, in particular at the moment that the specific (social) effects, which may raise ethical concerns, of these technologies become more clear, i.e. at the moment that these technologies become less experimental.

Following Beauchamp and Childress (2013), I propose to conceive of the conditions and the underlying moral principles as prima facie moral obligations, which means that they are morally obligatory unless there are overriding (moral) reasons not to follow them. The conditions are not only provisional in the sense that they may be overridden in specific circumstances, they are also open to improvement on the basis of experience with applying them. When we apply the principles, we might find out that they frequently lead to morally undesirable situations and this may be a reason to revise the conditions; this process of revision may be seen as a kind of (wide) reflective equilibrium process (Daniels 1996).

All in all, Table 3 is not intended as a checklist but rather as an argumentative ethical framework to decide about the acceptability of experimental technologies. To treat the conditions not as a checklist but rather as a framework for moral evaluation and discussion is also important in the light of the broader aim of this article. As I explained, the ethical framework developed wants to be a contribution to what Jasanoff has called technologies of humility. The suggestion that it would be possible to devise a ethical framework that answers all questions about the acceptability of social experiments with new technology without further discussion would amount to another technology of hubris. Instead we should acknowledge the important role of ethical and public debate when it comes to the introduction of new technologies into society. Much more can be said about how such debates should be organized, but that would be another article.

## Conclusions

I have argued that we can conceive of the introduction of experimental technologies into society as a social experiment. We will only experimentally and gradually find out some of the social consequences of these technologies. Adopting this perspective, I have then asked the question under which conditions such experiments are acceptable and I have developed an ethical framework for deciding so on basis of the four bioethical principles of non-maleficence, beneficence, respect for autonomy, and justice. The resulting ethical framework consists of sixteen conditions that are a specification of the four moral principles. These conditions are to be seen as prima facie moral obligations that are open to further specification for specific technologies and to revision in the light of new experiences. They are nevertheless a useful argumentative framework for evaluating the moral acceptability of experimental technology.

## Footnotes

1 See e.g. Beauchamp and Childress (2013). O'Neill (2003) argues that informed consent can be better understood in terms of non-maleficence. Even if she might be right that ‘informed consent’ is not only a specification of ‘respect for autonomy’ but also of ‘non-maleficence,’ it would seem an overstatement to suggest that it is not motivated by ‘respect for autonomy’ as well (even if there may be disagreement about how to understand the notion of ‘autonomy’).

2 Three of these can already be found in the Belmont Report (The National Commission for the Protection Of Human Subjects of Biomedical and Behavioral Research 1978). Only non-maleficence is not mentioned as a separate ethical principle (or rather it is subsumed under beneficence).

3 According to them, of these principles only informed consent may be waived in specific circumstances.

4 For the Nuremberg code, see for example "Trials of War Criminals before the Nuremberg Military Tribunals under Control Council Law No. 10", Vol. 2, pp. 181-182. Washington, D.C.: U.S. Government Printing Office, 1949. For the Common Rule, see http://www.hhs.gov/ohrp/humansubjects/guidance/45cfr46.html#46.111 retrieved 3 March 2015, and for the Helsinki Declaration, see http://www.wma.net/en/30publications/10policies/b3/, retrieved 3 March 2015. A document with the coding of these three codes is available from the author on request.

5 Arguably the line between non-maleficence and beneficence is not always sharp. I have understood non-maleficence here as relating to preventing any harm *from* the experiment; taking away existing harm or preventing harm that is caused by factors that are unrelated to the experiment is categorized as beneficence (cf. Beauchamp and Childress 2013: 151).

6 The first three are also covered by Beauchamp and Childress (2013) in their discussion about justice, but the fourth is not explicitly included, but it is arguable also an aspect of the more general principle of justice.

7 It is of course open to debate what the normative implications are (if any) of the fact that the content of codes has changed over time. My own suggestion would be that it is not a sign of moral relativism but rather should be interpreted as a form of moral learning that takes place by trying out principles in practice and finding out that these do or do not cover all relevant moral obligations, considered judgements or intuitions, not unlike John Dewey’s idea that moral principles are hypotheses to be tested out in new situations (e.g. Dewey 1922).
